# The implementation of enhanced recovery after surgery pathway in patients undergoing posterior thoracolumbar fusion for degenerative spinal deformity

**DOI:** 10.1186/s12891-023-06146-x

**Published:** 2023-01-13

**Authors:** Yi Yuan, Shuai-Kang Wang, Xin-Yi Chai, Peng Wang, Xiang-Yu Li, Chao Kong, Shi-Bao Lu

**Affiliations:** 1 Department of Orthopedics, No.6 Hospital, Beijing, 100007 China; 2 Department of Orthopedics, Xuanwu Hospital, Capital Medical University, No.45 Changchun Street, Xicheng District, Beijing, 10053 China; 3 National Clinical Research Center for Geriatric Diseases, Beijing, 10053 China; 4 Capital Medical University, Beijing, 10053 China

**Keywords:** Degenerative spinal deformity, Thoracolumbar fusion, Enhanced recovery after surgery, ERAS, Complications

## Abstract

**Purpose:**

The prevalence of degenerative spinal deformity (DSD) and the increased cost of correction surgery impose substantial burdens on the health care and insurance system. The aim of our study was to investigate the effects of the implementation of Enhanced Recovery After Surgery (ERAS) protocol on postoperative outcomes after complex spinal surgery.

**Methods:**

A retrospective analysis of prospectively established database of DSD was performed. The consecutive patients who underwent open correction surgery for degenerative spinal deformity between August 2016 and February 2022 were reviewed. We extracted demographic data, preoperative radiographic parameters, and surgery-related variables. The ERAS patients were 1:1 propensity-score matched to a historical cohort by the same surgical team based on age, gender, BMI, and number of levels fused. We then compared the length of hospital stay (LOS), physiological functional recovery, and the rates of complications and readmissions within 90 days after surgery between the groups.

**Results:**

There were 108 patients included, 54 patients in the ERAS cohort, and 54 patients matched control patients in the historical cohort. The historical and ERAS cohorts were not significantly different regarding demographic characteristics, comorbidities, preoperative parameters, operative time, and reoperation rate (*P* > 0.05). Patients in the ERAS group had significantly shorter postoperative LOS (12.0 days vs. 15.1 days, *P* = 0.001), average days of drain and urinary catheters placement (3.5 days vs. 4.4 days and 1.9 days vs 4.8 days, respectively), and lower 90-day readmission rate (1.8% vs. 12.9%, *P* = 0.027). The first day of assisted-walking and bowel movement occurred on average 1.9 days (2.5 days vs. 4.4 days, *P* = 0.001) and 1.7 days (1.9 days vs. 3.6 days, *P* = 0.001) earlier respectively in the ERAS group. Moreover, the rate of postoperative urinary retention (3.7% vs. 16.7%, *P* = 0.026) and surgical site infection (0% vs. 7.4%, *P* = 0.046) were significantly lower with ERAS protocol applied.

**Conclusions:**

Our study confirmed that the ERAS protocol was safe and essential for patients undergoing thoracolumbar deformity surgery for DSD. The ERAS protocol was associated with a shorter postoperative LOS, a lower rate of 90-day readmission, less rehabilitation discharge, and less postoperative complications.

## Introduction

Degenerative spinal deformity (DSD) occurs in individuals without pre-existing deformity and is the result of cumulative degenerative changes of spinal musculoskeletal and intervertebral discs that occur with aging [1]. Patients with degenerative spinal deformity often present with coexisting degenerative pathologies including spondylolisthesis, spinal stenosis, and degenerative disk disease, and they often complain of back or radicular pain and activity intolerance [2]. With an aging worldwide population, the number of people suffering from DSD increases yearly. Open-posterior thoracolumbar deformity surgery is a standard procedure for decompressing the spinal cord and nerve root, augmenting the posterior construct rigidity, and realigning the spine in the coronal and sagittal planes [3]. The prevalence of spinal correction surgery and the increase in hospitalization costs impose substantial burdens on the health care and insurance system [4]. Postoperative complications and readmission after major spine surgery have diminished patient satisfaction, linked to healthcare systems reimbursement. These findings suggest that a comprehensive perioperative management pathway is needed to reduce the length of hospital stay (LOS), the incidence of postoperative complications and readmission, and improve patient satisfaction.

The enhanced recovery after surgery (ERAS) protocol is an evidence-based, multidisciplinary perioperative management pathway that reduces the surgical stress responses, LOS and the incidence of complications in minimally-invasive spinal surgery [5–7]. DSD surgery is associated with major trauma, increased estimated blood loss and more prolonged rehabilitations [8, 9]. Multimodal perioperative management regimens should be applied to correction surgery to reduce stress response and achieve early recovery. However, the evidence for implementing the ERAS protocol on postoperative outcomes after complex spinal surgery remains limited. Therefore, this study aimed to identify the impact of the ERAS protocol on postoperative outcomes, including the LOS, postoperative complications, physiological functional recovery, and 90-day readmission in patients undergoing open correction surgery for DSD.

## Materials and methods

### Study design

We performed a retrospective analysis of a prospectively established database of DSD. The ethics committee of our hospital approved the study (permit data 2018.4.3; no. 2018086). We reviewed the consecutive patients who underwent open correction surgery for DSD between August 2016 and February 2022. Inclusion criteria included age greater than 50 years and open correction surgery for degenerative spinal deformity. The exclusion criteria were: 1) revision surgery; 2) concomitant cervical spine surgery; 3) non-contiguous segmental surgery; 4) drug treatment for cancer; 5) incomplete postoperative information; 6) cognitive impairment; 7) neoplasm, infective damage to the vertebral structure. Applying ERAS in clinical practice is a process of continuous learning and improvement. Although the ERAS protocol was initially introduced at our center in January 2019, the full implementation of the ERAS program began in July 2019. Therefore, the ERAS group consisted of patients who underwent surgery from August 2019 to January 2022, and the control group consisted of patients from August 2016 to December 2021.

### ERAS protocol

**Fig. 1:**
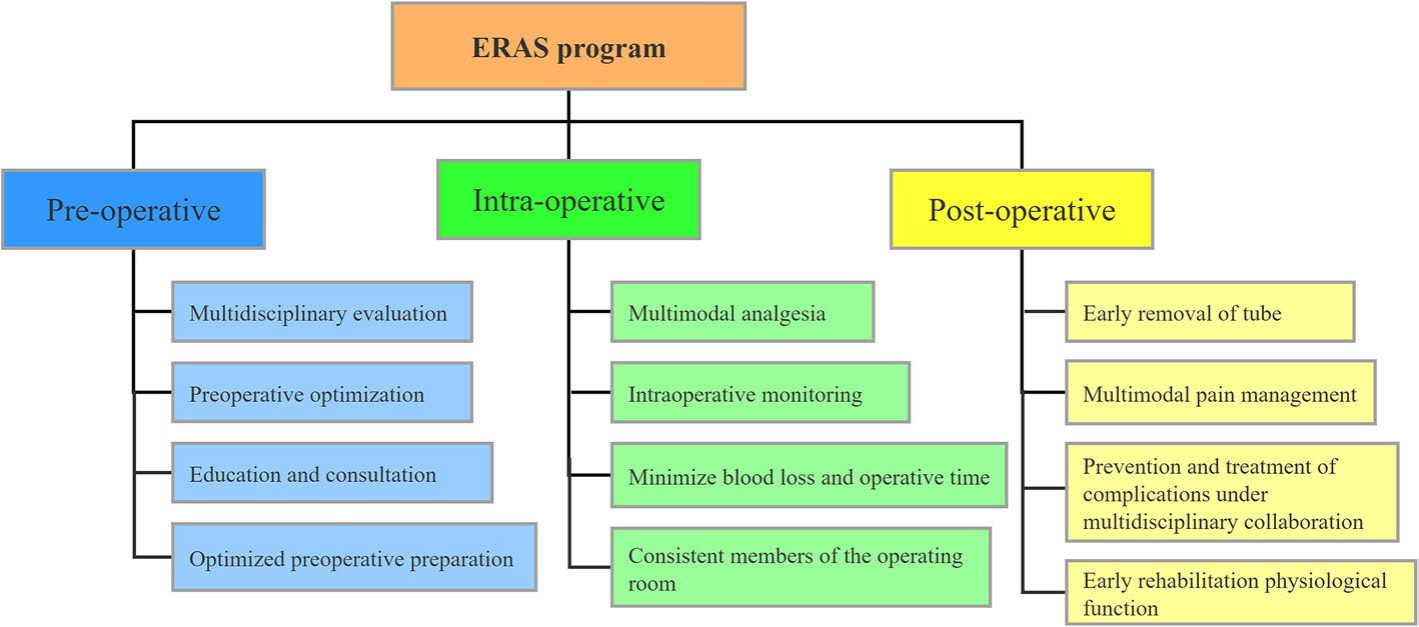
Our ERAS program

Our ERAS pathway includes preoperative, intraoperative, and postoperative multimodal management by a multidisciplinary team (Fig. 1). This protocol was implemented at our center after receiving institutional approval.

#### Multidisciplinary assessment and preoperative optimization

Preoperative multidisciplinary evaluation is the cornerstone of our ERAS program, which helps to predict the risk of perioperative adverse events and treat chronic disease. The spinal surgeon determines the severity of spinal deformities and the location of nerve compression and vertebral instability based on the symptoms and preoperative imaging. The spine surgery team then carries out the procedure plan. Based on the patient's laboratory test results and previous medical history, an internist evaluates the patient for severe chronic diseases, including coronary heart disease and hematologic diseases. Blood glucose and blood pressure levels are monitored and stabilized one week before surgery. Nutritionists perform preoperative nutritional evaluations using a nutritional screening scale and instruct patients to adjust their diet to improve preoperative nutrition. Psychologists assess the patient's mental status and provide psychological support if needed. A rehabilitation physician evaluates the patient's physical function and daily activity ability before surgery. Smokers are referred to a smoking cessation clinic one month before surgery, and opioid-dependent patients are referred to a chronic pain service center. Anesthesia-related risks are evaluated by senior anesthesiologists using the American Society of Anesthesiologists grade and the age-adjusted Charlson Comorbidity Index [10]. All evaluations results are collected preoperatively and used to guide surgical decision-making and perioperative interventions.

#### Education and consultation

Patients are educated on evaluating the degrees of pain using a numerical rating scale and Oswestry Disability Index. Expectation management includes patient education regarding the variability in pain improvement after fusion surgery. Patients are educated on the importance of physical rehabilitation and non-narcotic medication administration. Our center offers a multifaceted consulting service for patients with degenerative spinal deformity. The first-round consultation is conducted to determine patient expectations and inform patients regarding the surgical procedure and the possibility of perioperative adverse events. A geriatric consultation is provided for older patients. Patients with frailty and other systemic degenerative disorders (e.g., osteoporosis, Parkinson, and depression) are referred to corresponding clinics. A senior anaesthesiologist conducts a preoperative consultation regarding the adequate preoperative preparation and general anesthesia. All patients undergo live or online preoperative courses on upper and lower extremity strength exercises, lumbar back muscle exercises, and roll-over exercises in bed.

#### Optimized preoperative preparation

In patients undergoing elective DSD surgery, prolonged fasting predisposes to increased surgical stress response and surgery-induced insulin resistance. The preoperative preparation of the ERAS pathway requires cessation of clear liquids for two hours, and solid foods for eight hours before anesthesia. Other preoperative preparations include the administration of an oral carbohydrate-rich drink two hours before surgery and avoiding using mechanical bowel preparation. Establishing intraoperative blood pressure goals, maximum allowable blood loss and transfusion triggers are also essential in optimizing preoperative preparation.

#### Intraoperative management

The ERAS pathway includes multimodal analgesia, continuous monitoring, minimizing blood loss and operative time, and consistent members of operating room. Patients in the ERAS group were administered oral pregabalin two hours before surgery. All patients received general anesthesia with intravenous propofol and remifentanil according to weight and operation time. A mixture of 10 ml 2% lidocaine and 10 ml 1% ropivacaine was infiltrated around the surgical incision before incision and after skin closure. Intraoperative monitoring focuses on blood pressure, urinary volume, neurophysiological monitoring, and blood loss. Patients without contraindications were routinely given intravenous antifibrinolytics (tranexamic acid) to reduce blood loss. Applying topical hemostatic material and blood pressure control were also important interventions to control intraoperative bleeding. All DSD surgeries were performed by the same operating room staff, including a surgeon, anesthesiologist, and circulating nurse, contributing to reduced operation time. Unless extubation failed, the patients routinely returned to the ward after surgery.

#### Postoperative management

The post-operative ERAS protocol includes multimodal pain management, early removal of urinary tube and drainage tube, preventing and treating complications, and early recovery of physiological function. Multimodal pain management involves a combination of acetaminophen, steroids, gabapentin, pregabalin, cyclooxygenase-2 inhibitors, and neuraxial anesthesia with different mechanisms of action to reduce the use of opioids and optimize pain control. The urinary tube (if placed) was routinely removed within 72 h after surgery, except in rare cases. Unless complications occur, the drainage tube is removed within 96 h after surgery. From postoperative days 0 to 3, anti-vomiting drugs and gastric mucosal protective agents are routinely infused intravenously to alleviate adverse reactions in the gastrointestinal tract. Antithrombotic prophylaxis includes on-bed movement, early off-bed mobilization, lower extremity pneumatic pump application, and compression stocking placement. For patients with histories of hypercoagulable or thrombophilic clotting abnormalities, low molecular weight heparin is injected subcutaneously every 24 h for four to five days. Early recovery of physiological function involves improving physical function and early recovery of gastrointestinal function. All patients are encouraged to ambulate on postoperative day 1 with the assistance of nursing staff and start physical exercise from postoperative day 0 with the guidance of a rehabilitation physician. Other ERAS protocol items include early oral fluid and food intake after surgery, nutritional support, early intravenous fluids discontinuation, and oral administration of docusate suppository if no bowel movement occurs within 48 h after surgery.

### Data collection

From a prospectively established database, we extracted demographic data (age, gender, body mass index [BMI], payer status, comorbidities, chronic opioid use), preoperative radiographic parameters (lumbar lordosis [LL], sagittal vertical axis [SVA], pelvic tilt [PT], thoracic kyphosis [TK], pelvic incidence [PI] and pelvic incidence minus the lumbar lordosis angle [PI-LL], thoracolumbar kyphosis [TLK]), and surgery-related variables (operative time, operating room duration, estimated blood loss, number of fused levels, laminectomy, and interbody fusion). To control for differences in baseline data, ERAS patients were 1:1 propensity-score matched to a historical cohort by the same surgical team based on age, gender, BMI, and the number of levels fused. LOS was the primary outcome measure. Physiological functional outcome indicators such as time to first bowel movement, first ambulation, and days of urethral indwelling catheter were assessed as secondary outcome measures. We also compared the rates of complications and readmissions between the two groups within 90 days after surgery.

### Statistical analysis

Continuous variables were expressed as mean ± standard deviation and analyzed using the two-tailed Student’s t-test or Mann–Whitney U-test as appropriate. Categorical variables were expressed as frequencies with percentages and analyzed using Fisher’s exact or chi-square tests. All statistical analyses were performed using SPSS Statistics 25 (SPSS, version 22.0, Inc., Chicago, IL, USA). Statistical significance was set at p < 0.05.

## Results

There were 108 patients included in the study, 54 patients in the ERAS cohort (August 2019 to January 2022), and 54 patients matched to the historical cohort (August 2016 to December 2019). Age (70.1 ± 7.7 vs. 69.6 ± 8.5 *P* = 0.777) and gender (77.8% vs. 77.8% female, p = 1.000) were similar between cohorts. The historical and ERAS cohorts were not significantly different regarding demographic characteristics, comorbidities, or preoperative parameters (*P* > 0.05) (Table 1). Regarding intraoperative variables, there were no differences in the number of levels fused (7.0 ± 1.6 vs. 7.1 ± 1.6, *P* = 0.952), interbody fusion (2.2 ± 1.4 vs. 1.9 ± 0.9, *P* = 0.196), vertebral body osteotomy (20.3% vs 18.5%, *P* = 0.798), or laminectomy (2.9 ± 1.2 vs. 2.8 ± 1.2, *P* = 0.373) between the groups (Table 2). Patients in the ERAS group had longer operative time, without a statistically significant differences (363.9 ± 92.6 min vs. 333.0 ± 75.0 min, *P* = 0.059). The rates of patients with fusion to the sacrum (62.9% vs. 61.1%, *P* = 0.843) were similar between groups. Patients in the ERAS cohort had a lower estimated blood loss (825.7 ± 581.3 ml vs. 1181.7 ± 681.4 ml, *P* = 0.001).

**Table 1:** Comparison of demographic, clinical, and radiographic parameters between the enhanced recovery cohort and a matched historical cohort

Variables	ERAS cohort (*n* = 54)	Historical cohort (*n* = 54)	*P* Value
Female, n (%)	42 (77.8%)	42 (77.8%)	1.000
Age (y)	70.1 ± 7.7	69.6 ± 8.5	0.777
Weight (kg)	66.1 ± 12.2	67.7 ± 11.6	0.512
BMI (kg/m^2^)	25.9 ± 4.4	26.6 ± 3.6	0.355
*Payer status, n (%)*			0.540
Medicare	49 (90.7%)	47 (87.0%)	
Self-pay	5 (9.3%)	7 (13.0%)	
Smoker, n (%)	2 (3.7%)	5 (9.3%)	0.241
Drinker, n (%)	5 (9.3%)	3 (5.6)	0.462
ASA	2.1 ± 0.5	2.1 ± 0.3	0.832
*Co-Morbidities, n (%)*			
Charlson Comorbidity Index	3.0 ± 1.2	3.4 ± 1.1	0.200
Hypertension	20 (37.0%)	30 (55.6%)	0.054
Coronary heart disease	6 (11.1%)	7 (13.0%)	0.771
Diabetes disease	12 (22.2%)	15 (27.8%)	0.511
Mental disease	1 (1.8%)	0 (0%)	0.322
Digestive disease	1 (1.8%)	1 (1.8%)	1.000
Old cerebral infarction	5 (9.3%)	2 (3.7%)	0.242
Osteoporosis	21 (38.9%)	16 (29.6%)	0.315
Opioids consumption	3 (5.6%)	1 (1.8%)	0.315
Parkinsons disease	2 (3.7%)	5 (9.3%)	0.241
*Radiographic parameters*			
SVA (cm)	7.3 ± 5.3	6.3 ± 4.5	0.311
PI ( °)	50.7 ± 11.8	51.8 ± 11.1	0.640
PT ( °)	27.5 ± 14.0	26.4 ± 10.9	0.632
SS ( °)	22.3 ± 10.5	26.2 ± 10.7	0.062
TLK ( °)	24.0 ± 15.0	20.4 ± 17.2	0.243
TK ( °)	26.6 ± 12.2	27.1 ± 16.8	0.877
LL ( °)	23.2 ± 16.0	28.6 ± 16.7	0.086
PI-LL (°)	25.9 ± 16.6	20.8 ± 14.8	0.254

*BMI* body mass index, *SVA* sagittal vertical axis, *PI* pelvic incidence, *PT* pelvic tilt, *SS* sacral slope, *TLK* thoracolumbar kyphosis, *TK* thoracic kyphosis, *LL* lumbar lordosis, *PI-LL* pelvic incidence minus the lumbar lordosis angle. *P < 0.05

**Table 2:** Comparison of surgical characteristics between the enhanced recovery cohort and a matched historical cohort

Variables	ERAS cohort (*n* = 54)	Historical cohort (*n* = 54)	*P* Value
Number of levels fused	7.0 ± 1.6	7.1 ± 1.6	0.952
Interbody fusion	2.2 ± 1.4	1.9 ± 0.9	0.196
Laminectomy	2.9 ± 1.2	2.8 ± 1.2	0.373
Vertebral body osteotomy	11 (20.3%)	10 (18.5%)	0.798
Operative time (min)	363.9 ± 92.6	333.0 ± 75.0	0.059
Operating room duration(min)	435.9 ± 90.1	406.5 ± 70.9	0.075
Estimated blood loss (ml)	825.7 ± 581.3	1181.7 ± 681.4	0.001^*^
Fusion to the sacrum, n (%)	34 (62.9%)	33 (61.1%)	0.843

^*^P < 0.05

The total LOS in both groups was comparable; however, patients in the ERAS group had significantly shorter postoperative LOS (12.0 days vs. 15.1 days, *P* = 0.001). With regards to the postoperative physiological function, the first day of assisted-walking and bowel movement occurred on average 1.9 days (2.5 days vs. 4.4 days, *P* = 0.001) and 1.7 days (1.9 days vs. 3.6 days, *P* = 0.001) earlier with ERAS, respectively (Table 3). The average drain and urinary catheters placement days were significantly shorter in the ERAS cohort than in the historical cohort (3.5 days vs. 4.4 days and 1.9 days vs. 4.8 days, respectively). The rate of total complications was similar between the groups (22.2% vs. 33.3%, *P* = 0.198); however, fewer patients in the ERAS group had urinary retention (3.7% vs. 16.7%, *P* = 0.026) and surgical site infection (SSI) (0% vs. 7.4%, *P* = 0.046). There were no significant differences in other complications including deep venous thrombosis, pneumonia and hematoma (*P* > 0.05). Four patients in the pre-ERAS group were discharged to inpatient rehabilitation, while all patients in the ERAS group were discharged home. The 90-day readmission rate was significantly higher before the implementation of our ERAS program (12.9% vs. 1.8%, *P* = 0.027). Reoperation rates were similar between groups. Two patients in the EARS cohort underwent reoperation for hematoma. In the historical cohort, one patient underwent reoperation for SSI and another for hematoma.

**Table 3:** Comparison of clinical outcomes and physiological functional status between the enhanced recovery cohort and a matched historical cohort

Variables	ERAS cohort (*n* = 54)	Historical cohort (*n* = 54)	*P* Value
*LOS (day)*			
Total LOS	22.4 ± 7.7	24.1 ± 10.9	0.354
Preoperative LOS	10.3 ± 5.0	8.9 ± 4.7	0.149
Postoperative LOS	12.0 ± 4.5	15.1 ± 7.7	0.001^*^
Physiological functional status			
Foley discontinuation POD	1.9 ± 1.1	4.8 ± 2.0	0.001^*^
1st ambulation on POD	2.5 ± 1.4	4.4 ± 2.2	0.001^*^
1st bowel movement on POD	1.9 ± 0.7	3.6 ± 1.4	0.005^*^
Drain placement (day)	3.5 ± 1.1	4.4 ± 1.9	0.002^*^
*Complications, n (%)*	12 (22.2%)	18 (33.3%)	0.198
Myocardial infarction	0 (0%)	1 (1.8%)	0.315
Acute cerebral infarction	0 (0%)	1 (1.8%)	0.315
Urinary retention	2 (3.7%)	9 (16.7%)	0.026^*^
SSI	0 (0%)	4 (7.4%)	0.042^*^
Pneumonia	1 (1.8%)	4 (7.4%)	0.169
Hematoma	2 (3.7%)	1 (1.8%)	0.558
DVT	0 (0%)	1 (1.8%)	0.315
Urinary tract infection	1 (1.8%)	2 (3.7%)	0.558
Ileus	0 (0%)	3 (5.6%)	0.079
Nausea/vomiting	5 (9.3%)	6 (11.1%)	0.750
Delirium	1 (1.8%)	2 (3.7%)	0.558
Leakage of cerebrospinal fluid	0 (0%)	1 (1.8%)	0.315
Neurological deficit	0 (0%)	2 (3.7%)	0.153
The rate of readmission, n (%)	1 (1.8%)	7 (12.9%)	0.027^*^
*Discharge disposition, n (%)*			0.042^*^
Home	54 (100%)	50 (92.6%)	
Inpatient rehab	0 (0%)	4 (7.4%)	
The rate of reoperation, n (%)	2 (3.7%)	2 (3.7%)	1.000

*LOS* length of hospital stay, *POD* postoperative day, *SSI* surgical site infection, *DVT* deep venous thrombosis

## Discussion

Many previous studies described interventions to improve postoperative outcomes and satisfaction after spine surgery, including nutritional support [11], preoperative education, and early rehabilitation [12, 13]. These studies revealed significant reductions in postoperative complications. ERAS is an evidence-based perioperative care approach including multiple interventions [7]. There are various ERAS protocols for major surgical specialties, including spinal surgery; however, most existing protocols for spine surgery relate to minimally invasive and short-segment lumbar fusion. The safety and effectiveness of implementing the ERAS protocol in major surgery are worth considering. Furthermore, DSD surgery is often performed on older patients, who are more likely to have complications after spinal surgery [14]. In this retrospective study, we described the implementation of our ERAS protocol in DSD surgery. We found that ERAS patients had significantly shorter postoperative LOS, lower rates of postoperative complications (including urinary retention and SSI), lower rates of readmission, and less rehabilitation discharge.

Earlier clinical care pathways for fusion surgery were designed to reduce the length and variation of the inpatient stay by accelerating recovery [15]. In a retrospective study of 40 ERAS patients, Kim et al. reported a reduction in average LOS from 7.3 days to 4.5 days after thoracolumbar deformity surgery with the implementation of the ERAS protocol [16]. Dagal et al. compared 183 subjects in a traditional care group to 267 in an enhanced perioperative care group in a single academic spine surgery center and found that the ERAS group had a lower LOS and intensive care unit LOS than the pre-ERAS group (8.2 d vs. 6.1 d) [17]. In the current cohort, we found that ERAS protocol reduced postoperative LOS despite not changing the total LOS. We believe that this result was due to an emphasis on preoperative patient preparation, including a comprehensive assessment and optimization approach. We believe future studies on ERAS protocol should focus on shortening the preoperative assessment time and accelerating the preoperative preparation process. Lovecchio et al. conducted a retrospective cohort study and found that lower estimated blood loss (< 1200 mL), procedure end time before 15:00, and shorter operating room time were associated with shorter LOS [18]. This result highlighted the importance of a monitoring protocol, consistent surgical and anesthesia teams, and minimizing intraoperative bleeding.

Physiological functions include recovery of bowel function, voluntary urination, improvement of performance and nutritional status, and drainage reduction [19]. Early recovery of physiological function helps reduce time in bed and the incidence of postoperative complications. Our ERAS strategy substantially facilitated the early recovery of physiological functions and reduced the incidence of postoperative complications after major spine surgery. Previous studies drew inconsistent conclusions regarding the impact of ERAS protocol on complications after major spine surgery [20]. Porche et al. [19]and Dagal et al. [17]found no differences between the ERAS and Pre-ERAS groups regarding postoperative complications after spinal surgery. We observed a lower rate of postoperative SSI in the ERAS group. Several reasons might explain these discrepancies. First, patients with malnutrition and a long history of heavy smoking were identified and transferred to the corresponding clinic after preoperative assessment. Second, strict glycemic control and early drainage tube removal were associated with a lower incidence of SSI after spine surgery. Inadequate nutrition and poor glycemic control have been identified as risk factors for wound-related complications [21–23]. As expected, we found that patients in the ERAS cohort had a significantly lower rate of urinary retention than the historical cohort. This result is consistent with the previously reported literature by Adeyemo et al. [24]. Early ambulation and removal of the urinary catheter may account for the ERAS group’s lower urinary retention rate (3.7% vs. 16.7%).

Our ERAS program included inpatient physical rehabilitation and evaluation of physical function prior to discharge. We believe these interventions are the major drivers for the high rate of home discharge. Unplanned readmission is associated with poor satisfaction and additional costs among patients with spine surgery [25, 26]. The 90-day readmission was lower in the ERAS cohort than in the historical cohort. The association between enhanced recovery care and reduced readmission rates was demonstrated in previous studies, including short-segment and long-segment fusion surgery [24, 27]. While this finding was not an objective of the study, our research reinforces the role of ERAS in reducing patient hospitalization costs.

There are several limitations in the present study. First, this was a retrospective study, and the two cohorts were selected from two consecutive periods. However, given the complexity of the ERAS process and the necessity of education, prospective controlled trials are challenging. Second, our population size was relatively small, and all patients were from a single center. Multicenter studies with large sample sizes may contribute to developing ERAS protocol and determination of important differences between groups. Moreover, our follow-up period was 90 days, which was insufficient to detect the long-term effects of ERAS on patients undergoing surgery for degenerative spinal deformities. At last, some clinical and patients-reported outcomes were not included in our analysis, including treatment cost, postoperative pain control and the dependence in daily activities (which were also of concern to patients and surgeons). Despite these shortcomings, our study has some notable strengths. To our knowledge, this is the first study to describe the implementation of the ERAS protocol in patients with DSD. We enrolled consecutive patients with homogeneity and compared several surgical outcomes between the groups. Additionally, by matching patients in the history cohort by age, gender, BMI, and the number of fused levels, confounders were less likely to affect the comparison between the groups.

## Conclusions

The ERAS protocol is safe and effective for patients undergoing open-thoracolumbar deformity surgery for DSD. The ERAS protocol is associated with a shorter postoperative LOS, a lower rate of 90-day readmission and a higher rate of discharge to home, and fewer postoperative complications. The protocol could facilitate early recovery of physiological functions, including shortening the time in bed, early removal of drainage and urinary tube, and enhanced recovery of bowel movement. The establishment of a preoperative fast-track assessment process can reduce the preoperative LOS and improve the generalizability of ERAS. Future studies should focus on the ability of perioperative management to maximize its potential to improve patient satisfaction and reduce hospitalization costs.

## Data Availability

The underlying data supporting the results of this study could be obtained by contacting the corresponding author.

## Abbreviations

DSD: Degenerative spinal deformity
LOS: Length of hospital stay
ERAS: Enhanced recovery after surgery
EBL: Estimated Blood Loss
LL: Lumbar lordosis
SVA: Sagittal vertical axis
PT: Pelvic tilt
TK: thoracic kyphosis
PI: Pelvic incidence
PI-LL: Pelvic incidence minus the lumbar lordosis angle
TLK: Thoracolumbar kyphosis
BMI: Body Mass Index
